# Introduction to the National Epidemiologic Survey on Alcohol and Related Conditions

**Published:** 2006

**Authors:** Bridget F. Grant, Deborah A. Dawson

**Affiliations:** Bridget F. Grant, Ph.D., Ph.D., is chief of, and Deborah A. Dawson, Ph.D., is senior clinician in the Laboratory of Epidemiology and Biometry, Division of Intramural Clinical and Biological Research, at the National Institute on Alcohol Abuse and Alcoholism, Bethesda, Maryland

In 2001/2002, the National Institute on Alcohol Abuse and Alcoholism (NIAAA) conducted the National Epidemiologic Survey on Alcohol and Related Conditions (NESARC), the largest and most ambitious comorbidity study ever conducted. In addition to an extensive battery of questions addressing present and past alcohol consumption, alcohol use disorders (AUDs), and utilization of alcohol treatment services, NESARC included similar sets of questions related to tobacco and illicit drug use (including nicotine dependence and drug use disorders). Furthermore, NESARC contained questions that operationalized the criteria set forth in the American Psychiatric Association’s *Diagnostic and Statistical Manual of Mental Disorders, Fourth Edition* (DSM–IV) for the following psychiatric disorders:

- Five mood disorders (major depressive disorder, bipolar I and bipolar II disorders, dysthymia, and hypomania)
- Four anxiety disorders (panic with and without agoraphobia, social phobia, specific phobia, and generalized anxiety)
- Seven personality disorders (avoidant, dependent, obsessive–compulsive, paranoid, schizoid, histrionic, and antisocial disorders).

The unprecedented sample size of NESARC (*n* = 43,093) made it possible to achieve stable estimates of even rare conditions. Moreover, its oversampling of Blacks and Hispanics as well as the inclusion of Hawaii and Alaska in its sampling frame yielded enough minority respondents to make NESARC an ideal vehicle for addressing the critical issue of race and/or ethnic disparities in comorbidity and access to health care services.

NESARC’s diagnostic classifications were based on the Alcohol Use Disorder and Associated Disability Interview Schedule–DSM–IV Version (AUDADIS–IV), a state-of-the-art, semistructured diagnostic interview schedule designed for use by lay interviewers. The reliability and validity of this instrument have been documented in a wide range of international settings, using both general population and clinical samples (for an extensive list of publications on reliability and validity, see the data notes section of the NESARC Web site).

## General Characteristics and Purpose of NESARC

The NESARC longitudinal survey consists of a first wave (Wave 1), which was conducted from 2001 to 2002, and a second wave (Wave 2), which was conducted from 2004 to 2005. The NESARC sample represents the civilian, noninstitutionalized adult population of the United States, including residents of the District of Columbia, Alaska, and Hawaii. It includes people living in households, military personnel living off base, and people residing in the following group quarters: boarding or rooming houses, nontransient hotels and motels, shelters, facilities for housing workers, college quarters, and group homes. All potential NESARC respondents were informed in writing about the nature of the survey, the statistical uses of the survey data, the voluntary aspect of their participation, and the Federal laws that rigorously provide for the confidentiality of identifiable survey information. Respondents who consented to participate after receiving this information were interviewed. The research protocol, including informed-consent procedures, received full ethical review and approval from the U.S. Census Bureau and the U.S. Office of Management and Budget.

Data were collected in face-to-face, computer-assisted personal interviews conducted in respondents’ homes. The NESARC response rate was 81 percent.

**Topics Covered in NESARC**

NESARC studies the occurrence of more than one psychological disorder or substance use disorder in the same person, using definitions from the *Diagnostic and Statistical Manual of Mental Disorders, Fourth Edition* for these disorders. The survey collects demographic information on the people interviewed as well as the following types of information about them:

**Alcohol Use**

- Initiation of use
- Consumption patterns (frequency of drinking and of intoxication, amounts consumed) over the last 12 months and throughout the lifetime
- Circumstances surrounding drinking
- Beverage-specific consumption
- Alcohol experiences (effects and consequences of drinking, development of tolerance, attempts to stop drinking)
- Experiences with treatment for alcohol abuse and dependence
- Family history of alcoholism

**Tobacco Use**

- Initiation of use
- Consumption patterns (amount, frequency, duration)
- Consequences of tobacco use
- Attempts to stop using tobacco

**Use of Other Medications and Drugs**

- Sedatives, tranquilizers, painkillers, stimulants
- Marijuana
- Cocaine, hallucinogens, inhalants, heroin
- Other medications and drugs (psychoactive drugs, steroids)
- Initiation of use
- Usage patterns (during the last 12 months and across the lifetime)
- Consequences of use
  – Physical and mental effects
  – Signs of dependency
  – Attempts to stop or cut down on use
- Use of treatment
- Family history of substance use and abuse

**Psychological Disorders**

- Major depression
- Low mood (dysthymia)
- Mania and hypomania (a mild degree of mania)
- Panic disorders (with or without agoraphobia)
- Social phobia
- Specific phobias
- Generalized anxiety disorder
- Personality disorders (such as antisocial personality disorder)

**Family History**

- Of drug use
- Of major depression
- Of personality disorders

**Gambling**

**Medical Conditions/Victimization**

The major purposes of the Wave 1 and Wave 2 NESARC are to:

- Determine the prevalence, incidence, stability, and recurrence of AUDs and their associated disabilities in the general U.S. population.
- Estimate the magnitude of health disparities in AUDs and their associated disabilities among population subgroups defined by gender, race/ethnicity, disability, sexual orientation, age, and socioeconomic status.
- Estimate the size, characteristics, and changing nature of populations of special concern, including alcohol abusers and other people in the general population who are impaired or affected by the use of alcohol (e.g., those engaging in binge drinking or impaired driving).
- Estimate changes in AUDs and their associated disabilities over time, and identify factors associated with the natural history of AUDs.
- Determine the number of people receiving alcohol treatment through various treatment programs and services, including those not otherwise represented in surveys of treatment facilities; measure the unmet need for alcohol treatment services; and identify barriers to seeking treatment.
- Determine the associations between AUDs and their major physical and mental disabilities, differentiating drug-induced disorders from those reflecting true, independent mental conditions.
- Determine the boundaries between safe and hazardous drinking levels and patterns for various types of AUDs and their associated medical, social, and psychological sequelae.

## NESARC Design

The Census Supplementary Survey (C2SS) formed the sampling frame for the household portion of the NESARC sample. Of the approximately 2,000 C2SS primary sampling units (PSUs), which represented all 3,142 counties and county equivalents in the United States, 655 PSUs were selected with certainty because of their size (a population of 250,000 or more in 1996). These were designated as self-representing (SR) PSUs. The remaining PSUs were stratified within each State by sociodemographic characteristics. Two PSUs were selected from each stratum with probability proportional to size, yielding 254 additional PSUs that were designated as non-self-representing (NSR). To prevent possible respondent disclosure, the resulting 401 SR and 254 NSR PSUs were subsequently collapsed into 305 SR and 130 NSR PSUs. Within sample PSUs, households were systematically selected, and one adult respondent age 18 or older was selected at random from each sample household.

**Accessing the NESARC Data**

To access the NESARC data, go to: http:niaaa.census.gov/data.html.

From this site you can choose to:

- Read instructions for unzipping data files and running SAS programs.
- Download data (for Unix or PC Win/Zip).
- Download a SAS program file for converting the ASCII file into a SAS data set.

The Census 2000 Group Quarters Inventory formed the sampling frame for the group quarters portion of the NESARC sample. Individuals were randomly selected from a systematic sample of group quarters in these PSUs.

NESARC oversampled Blacks and Hispanics at the design phase of the survey, increasing the representation of Black households from 12.3 percent to 19.1 percent and the representation of Hispanic households from 12.5 percent to 19.3 percent. In addition, NESARC oversampled young adults ages 18–24 at the household level at a rate of 2.25 to 1. Again, one sample adult was randomly selected for interview in each household.

The NESARC sample was weighted to adjust for nonresponse at the household and person levels, the selection of one person per household, and oversampling of young adults, Hispanics, and Blacks. Once weighted, the data were adjusted to be representative of the U.S. population for various sociodemographic variables, including region, age, sex, race, and ethnicity, based on the 2000 Decennial Census.

## Availability of Wave 1 and Wave 2 NESARC Data

Data from Wave 1 of NESARC are available for downloading on the NESARC Web site at http://niaaa.census.gov. The Web site also has an online copy of the Wave 1 instrument, including flashcards containing response categories that were shown to the respondents, a full data tape codebook, a program to read the data into a SAS file, details of survey methodology, and a list of publications based on Wave 1 NESARC. Each respondent on the data file has been assigned a unique identifier. After Wave 2 NESARC data have been collected, cleaned, and placed on the Web site for public use, this identifier will be used to link the data from Waves 1 and 2.

Wave 2 NESARC differs from Wave 1 in several important ways:

- Whereas Wave 1 focused on the respondents’ entire lifetime up to the point of the interview (distinguishing the past year and the period prior to the past year), Wave 2 focuses only on the period since the Wave 1 interview (again distinguishing the past year from the period since the last interview but prior to the past year).
- Wave 2 adds questions for classifying several additional mental disorders, including post-traumatic stress disorder; attention deficit–hyperactivity disorder; and narcissistic, borderline, and schizotypal personality disorders.
- Wave 2 includes questions designed to measure sexual orientation, adverse childhood events (e.g., sexual abuse), childhood and partner abuse (physical, sexual, and psychological), social integration, and acculturation.
- Wave 2 adds numerous questions addressing perceived experiences of discrimination on the basis of gender, race/ethnicity, disability, sexual orientation, and weight.

## Summary of Findings

This issue of *Alcohol Research & Health* contains six articles based on data obtained in the Wave 1 NESARC. These articles illustrate just a few of the many diverse applications of the NESARC data, covering topics such as trends in the prevalence of AUDs over time; comorbidity of AUDs with other drug use disorders, mood and anxiety disorders, and personality disorders; prevalence and correlates of recovery from alcohol dependence; and prevalence and trend estimates for driving after drinking. Brief summaries of those articles follow.

### The 12-Month Prevalence and Trends in DSM–IV Alcohol Abuse and Dependence: United States, 1991–1992 and 2001–2002

This article describes the first trend analysis of DSM–IV AUDs ever conducted in the United States over the past decade. Its purpose was to present nationally representative data on the prevalence of 12-month DSM–IV alcohol abuse and dependence in 2001–2002 and to examine changes in alcohol abuse and dependence between 1991–1992 and 2001–2002. This analysis found that in 2001–2002, AUDs afflicted 17.6 million adult Americans. Abuse and dependence were more common among men and younger respondents than among women and older respondents. The prevalence of abuse was greater among Whites than among Blacks, Asians, and Hispanics. The prevalence of dependence was higher among Whites, Native Americans, and Hispanics than among Asians. Between 1991–1992 and 2001–2002, the prevalence of abuse increased while the prevalence of dependence declined. Increases in the rates of alcohol abuse were observed among men, women, and young Blacks and Hispanics, whereas the rates of dependence rose among men, particularly Asian men, and young Black women. This study underscores the need to continue monitoring prevalence and trends of AUDs and to design culturally sensitive prevention and intervention programs.

### Comorbidity Between DSM–IV Alcohol and Specific Drug Use Disorders in the United States: Results From the National Epidemiologic Survey on Alcohol and Related Conditions

This study provides detailed data on the homotypic^1^ comorbidity of AUDs and 25 different drug use disorders and confirms the high levels of association seen in previous studies based on lifetime measures. Prevalences were 7.35 percent for AUDs only, 0.90 percent for drug use disorder only, and 1.10 percent for comorbid alcohol and drug use disorders. Sociodemographic and psychopathologic correlates of individuals in these three groups were quite different, with the drug use disorder and comorbid groups more likely to be young, male, never married, and of lower socioeconomic status than the AUD-only group. Associations between current AUDs and 25 specific drug use disorders were generally positive and statistically significant. The 12-month prevalence of treatment-seeking was 6.06 percent for those with an AUD only, 15.6 percent for those with a drug use disorder only, and 21.8 percent for those with comorbid alcohol and drug use disorders. The findings of this study demonstrate the need for continuing efforts to integrate alcohol and drug treatment services.

### Prevalence and Co-Occurrence of Substance Use Disorders and Independent Mood and Anxiety Disorders: Results From the National Epidemiologic Survey on Alcohol and Related Conditions

This study was the first to distinguish independent from drug-induced DSM–IV mood and anxiety disorders and to examine their current co-occurrence with DSM–IV alcohol and drug use disorders in the United States. Drug use disorders and major mood and anxiety disorders that develop independently of acute intoxication and withdrawal are among the most prevalent psychiatric disorders in the United States. Associations between most drug use disorders and independent mood and anxiety disorders were overwhelmingly positive and significant, strongly suggesting that treatment for a comorbid mood or anxiety disorder should not be withheld from people with drug use disorders. The results also underscore the importance of ongoing development of improved treatment for people who meet criteria for two or more disorders.

### Co-Occurrence of 12-Month Alcohol and Drug Use Disorders and Personality Disorders in the United States: Results From the National Epidemiologic Survey on Alcohol and Related Conditions

This is the first study of the co-occurrence of DSM–IV alcohol and drug use disorders and personality disorders in the United States. It found that 28.6 percent of people with a current AUD had at least one personality disorder, as did 47.7 percent of those with a current drug use disorder. Further, 16.4 percent of people with at least one personality disorder had a current AUD, and 6.5 percent had a current drug use disorder. Overall, alcohol and drug use disorders were most strongly related to antisocial, histrionic, and dependent personality disorders. Associations between obsessive–compulsive, histrionic, schizoid, and antisocial personality disorders and specific alcohol and drug use disorders were significantly stronger among women than among men, whereas the association between dependent personality disorder and drug dependence was significantly greater among men than among women.

The finding that the co-occurrence of personality disorders with alcohol and drug use disorders is pervasive in the U.S. population highlights the need for further research on the underlying structure of these disorders and the treatment implications for patients with comorbidity of these disorders.

### Recovery From DSM–IV Alcohol Dependence: United States, 2001–2002

This was the first study of the prevalence and correlates of recovery from DSM–IV alcohol dependence in the U.S. general population that distinguished asymptomatic risk drinkers from low-risk drinkers and recent from stable recovery. This study examined people who met the criteria for DSM–IV alcohol dependence prior to the year immediately preceding the NESARC interview. Only 25.5 percent of these people had ever received treatment. The analysis found that:

- 25.0 percent of these people were still classified as dependent in the year before the NESARC interview.
- 27.3 percent were classified as being in partial remission.
- 11.8 percent were asymptomatic risk drinkers who demonstrated a pattern of drinking that put them at risk of relapse.
- 17.7 percent were low-risk drinkers.
- 18.2 percent were abstainers.

Factors associated with recovery included age, gender, marital status, education, interval since onset, severity, age at onset of dependence, tobacco and other drug use, and personality disorder. The analysis revealed that there are substantial levels of recovery from alcohol dependence. Information on factors associated with recovery should be used to improve the prospects for treatment.

### Twelve-Month Prevalence and Changes in Driving After Drinking: United States, 1991–1992 and 2001–2002

Drinking and driving has been identified as one of the most important contributors to motor vehicle fatalities, yet little is known about how driving-after-drinking rates have changed over the years. This article addresses this gap in public health knowledge by examining changes in the prevalence of driving after drinking in the United States between 1991–1992 and 2001–2002. Overall, the prevalence of driving after drinking was 2.9 percent in 2001–2002, corresponding to approximately 6 million U.S. adults. This rate was about three-quarters of the rate observed in 1991–1992 (3.7 percent), reflecting a 22-percent reduction. Generally, the male–female differentials in the rate of driving after drinking decreased over the past decade. However, the gender ratios increased substantially for underaged youth, reflecting the sharp decrease in prevalence of driving after drinking among 18- to 20-year-old women. Constant and emerging subgroups at high risk for drinking and driving included Whites, Native Americans, men, underaged youth, and young adults ages 21 to 25. The results of this study highlight the need to strengthen existing prevention and intervention efforts and to develop new programs for the prevention of drinking and driving.

## Summary

The articles included in this issue of *Alcohol Research & Health* represent only a small sample of the research that has been conducted using the 2001–2002 NESARC, but they demonstrate the range and dimensions of its data and some of the important issues to which these data may be applied. With the completion of Wave 2, NESARC will represent an unprecedented source of information on the natural history and comorbidity of AUDs and associated disabilities which will continue to set the standard for survey methodology, statistical analysis, and psychiatric epidemiology.

## Footnotes

1 Homotypic comorbidity refers to the co-occurrence of disorders within a certain diagnostic grouping (for example, dependence on sedatives in addition to an AUD). Heterotypic comorbidity refers to the co-occurrence of two disorders from different diagnostic groups (such as an AUD and major depression).

## GLOSSARY

Non-self-representing: A term applied to *primary sampling units* that are randomly selected or selected with some known probability other than certainty for inclusion into a sample.
Oversampling: A sampling technique used to augment numbers of participants from certain subgroups of interest, for whom *simple random sampling* would not result in a large enough number of cases to yield reliable estimates.
Primary sampling units: Comprehensive, mutually exclusive categories (often geographic areas, such as metropolitan statistical areas or counties) containing all persons or units of interest, usually identified in the first stage of a multistage sampling design.
Self-representing: A term applied to *primary sampling units* that are selected with certainty into the sample, usually because of their size.
Simple random sampling: A method of selecting samples in which each person, unit, or element of the sample has an equal probability of being selected.
Stratification: The classification of all persons, units, or elements of interest into comprehensive, mutually exclusive categories (strata).

